# Supplementary material for: Anti-Fibrotic Potential of Angiotensin (1-7) in Hemodynamically Overloaded Rat Heart
Source: Int J Mol Sci. 2023 Feb 9;24(4):3490. doi: 10.3390/ijms24043490 (PMC9967643; doi:10.3390/ijms24043490)
Supplement: Supplementary file 1 [file ijms-24-03490-s001.zip › ijms-2163807-supplementary.pdf]

SMAD2/3

LV

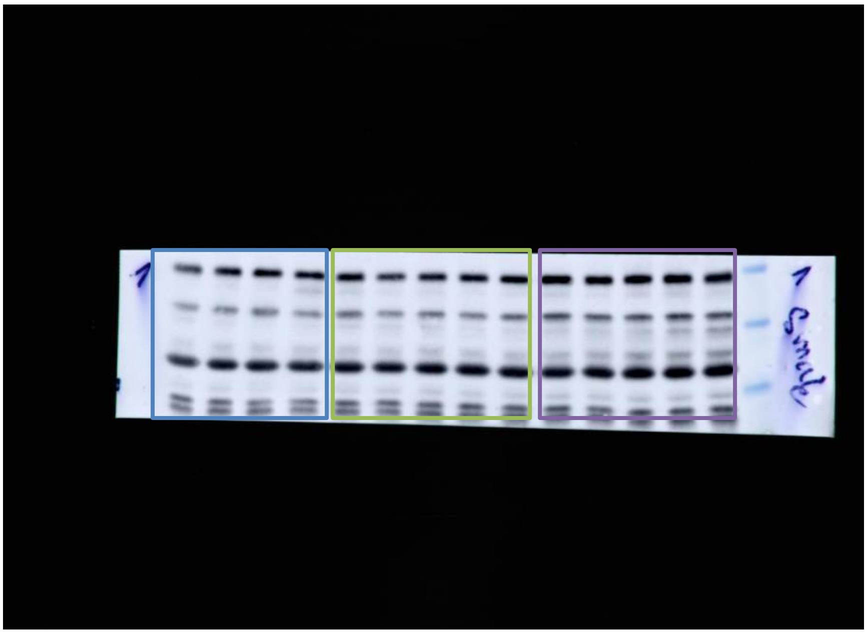

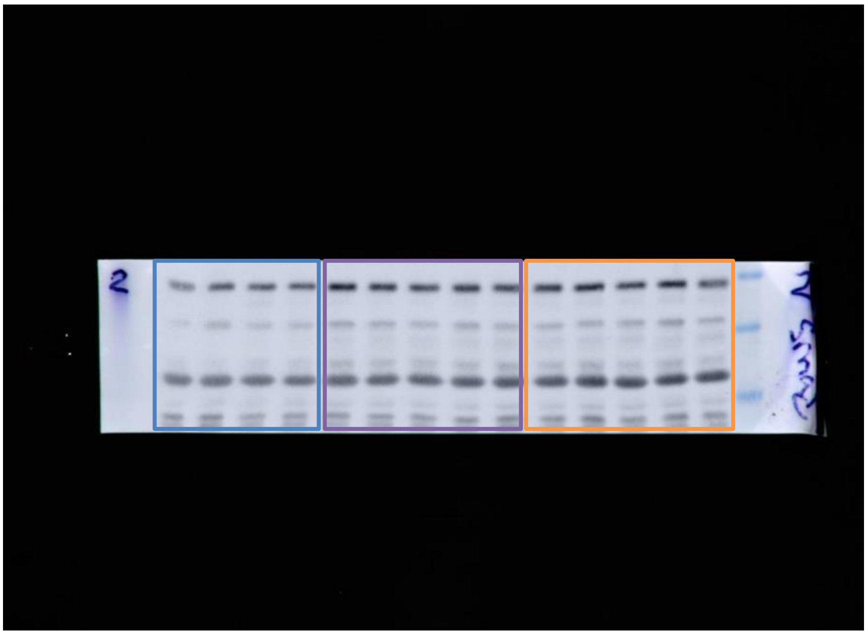

blue frame - HSD ACF
green frame - TGR ACF
violet frame - TGR(A1-7)3292
orange frame - TGR(A1-7)3292 ACF

RV

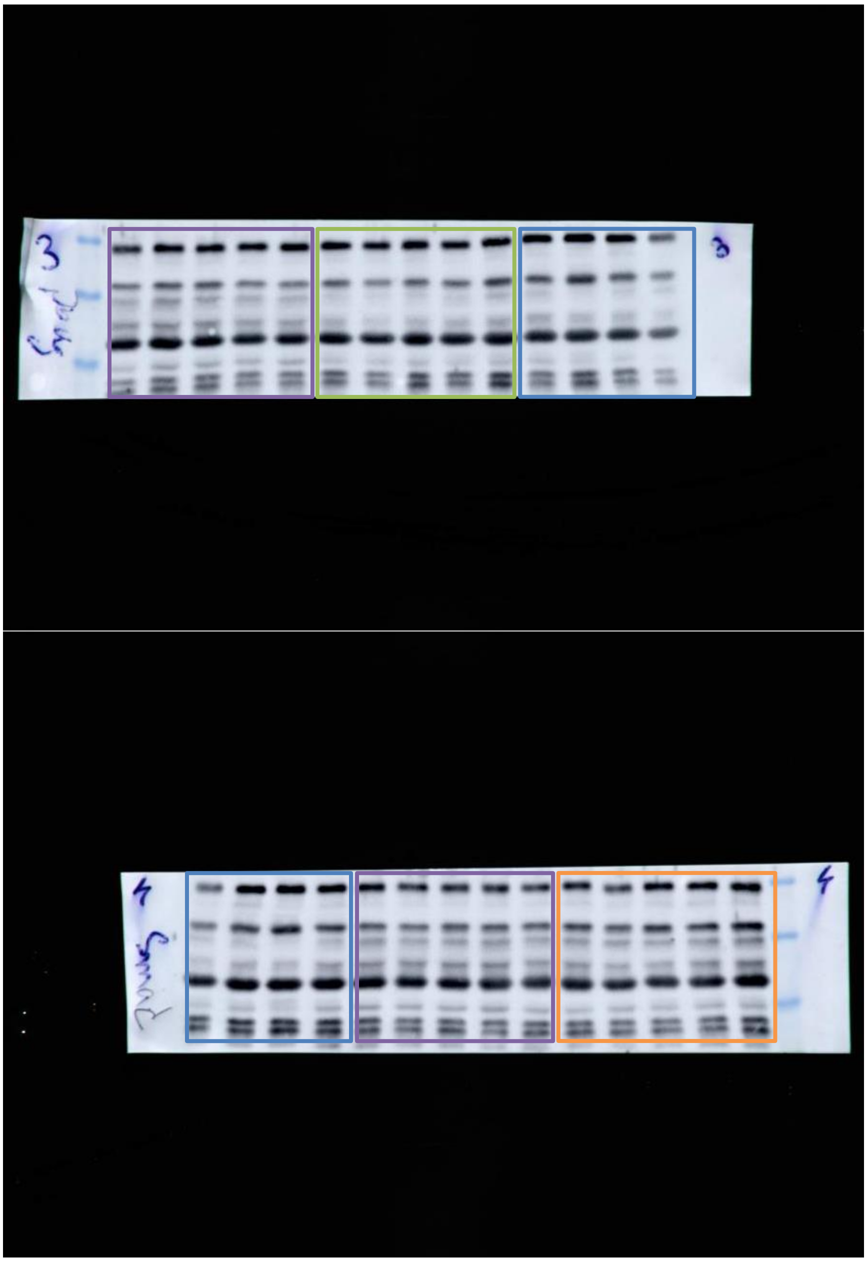

blue frame - HSD ACF
green frame - TGR ACF
violet frame - TGR(A1-7)3292
orange frame - TGR(A1-7)3292 ACF

PKCδ

LV

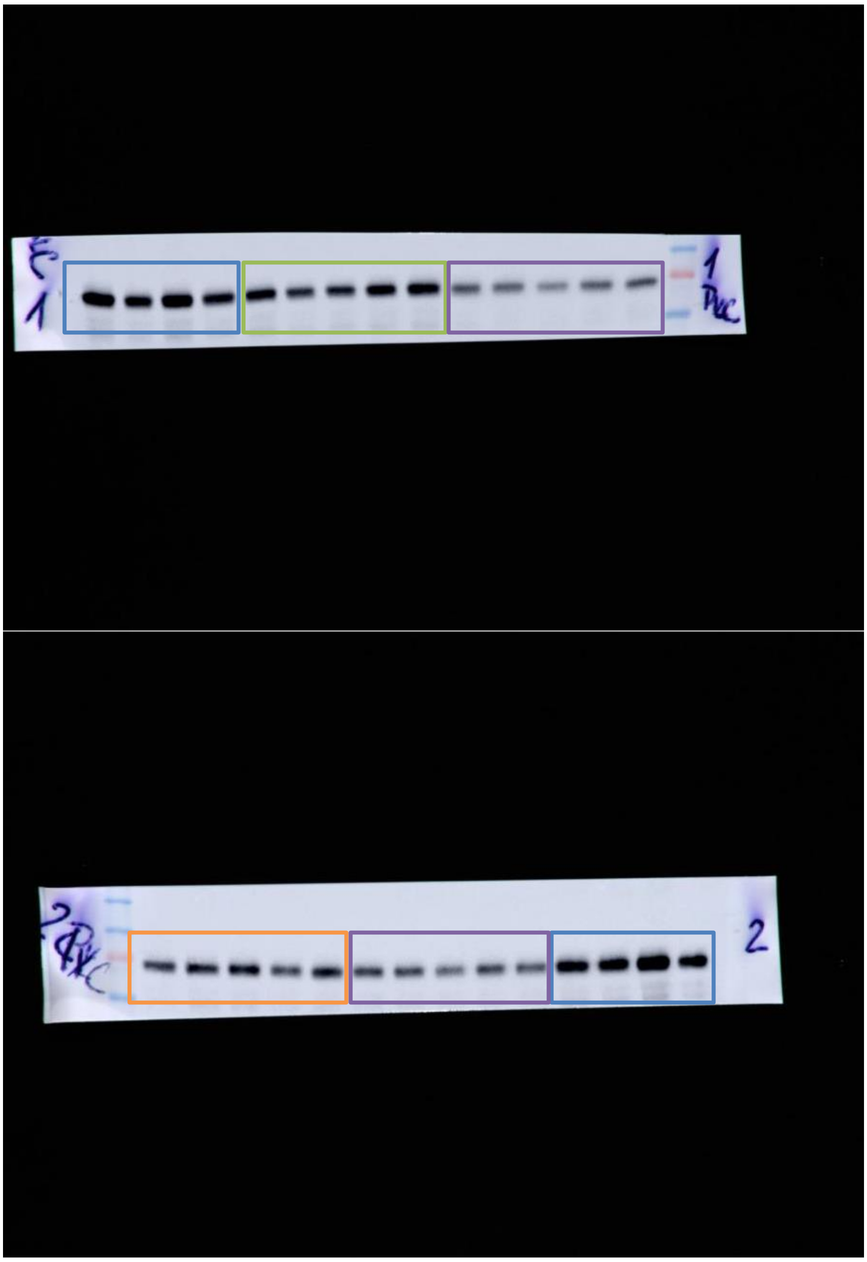

blue frame - HSD ACF
green frame - TGR ACF
violet frame - TGR(A1-7)3292
orange frame - TGR(A1-7)3292 ACF

RV

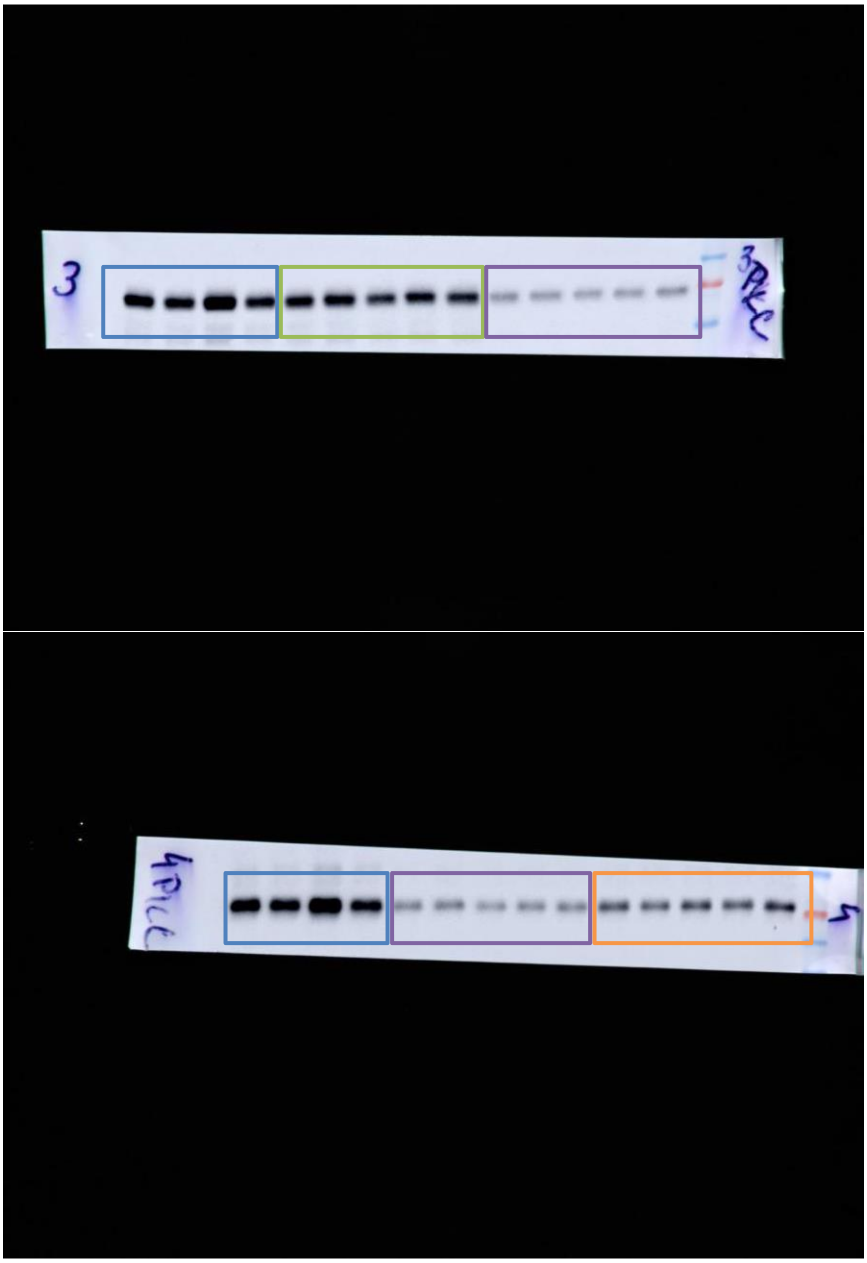

blue frame - HSD ACF
green frame - TGR ACF
violet frame - TGR(A1-7)3292
orange frame - TGR(A1-7)3292 ACF

PKCα

LV

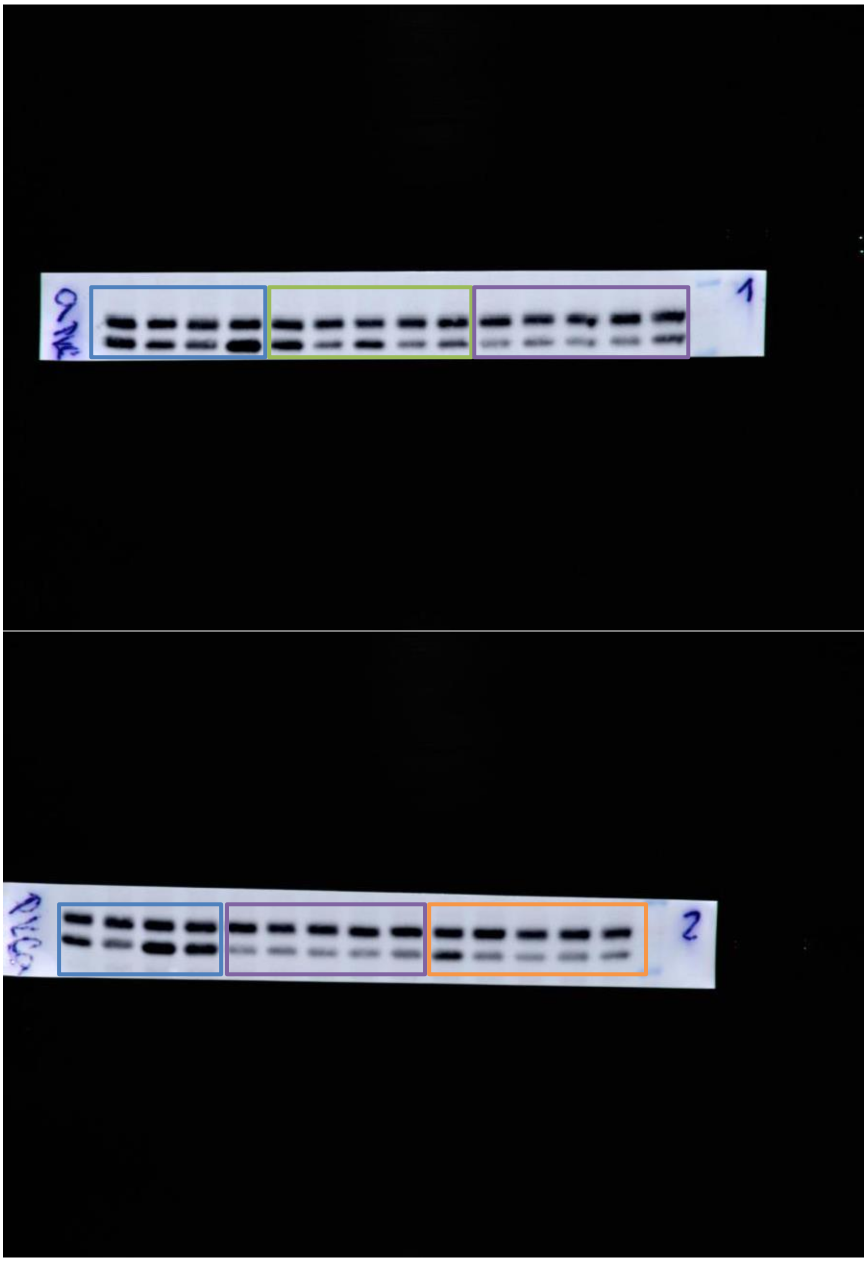

blue frame - HSD ACF
green frame - TGR ACF
violet frame - TGR(A1-7)3292
orange frame - TGR(A1-7)3292 ACF

RV

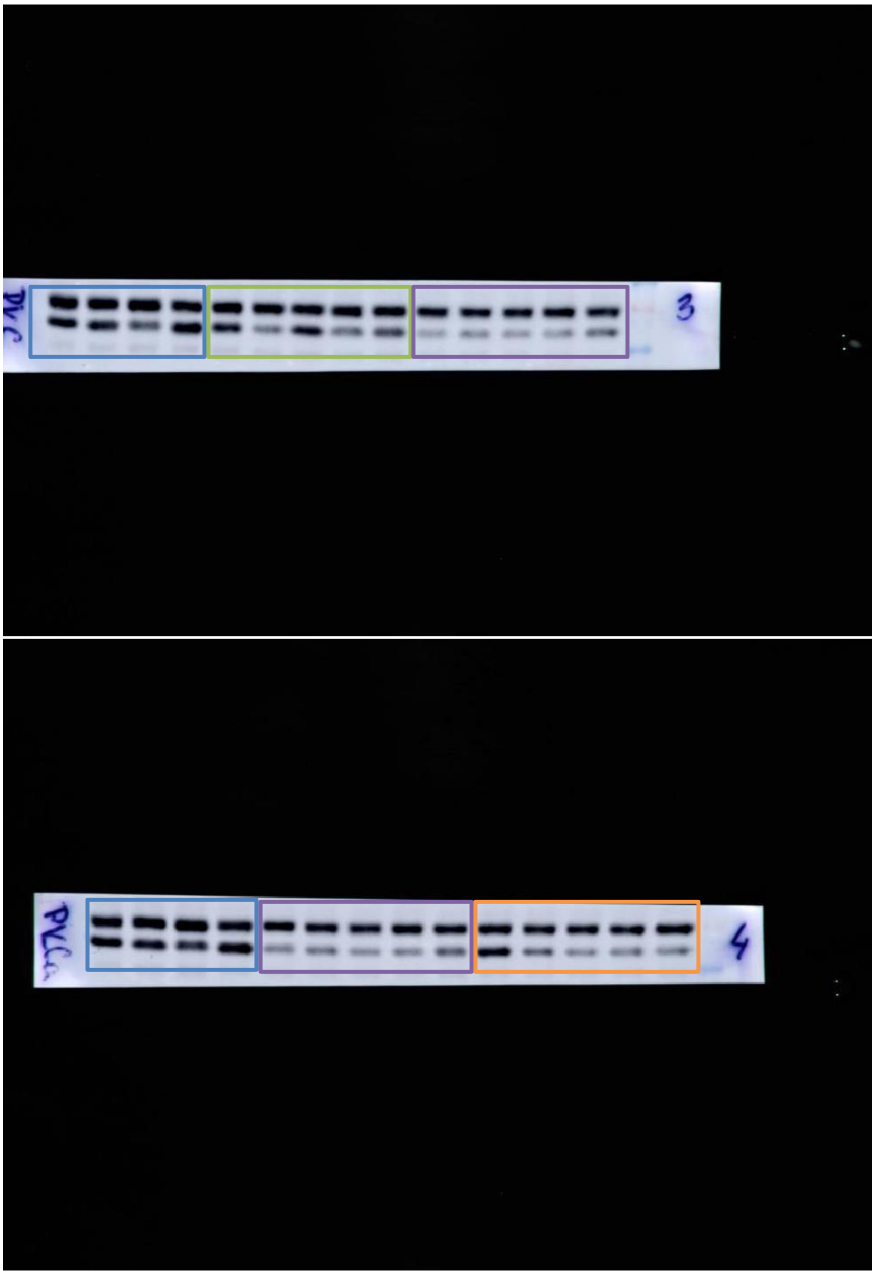

blue frame - HSD ACF
green frame - TGR ACF
violet frame - TGR(A1-7)3292
orange frame - TGR(A1-7)3292 ACF

PKCε

RV

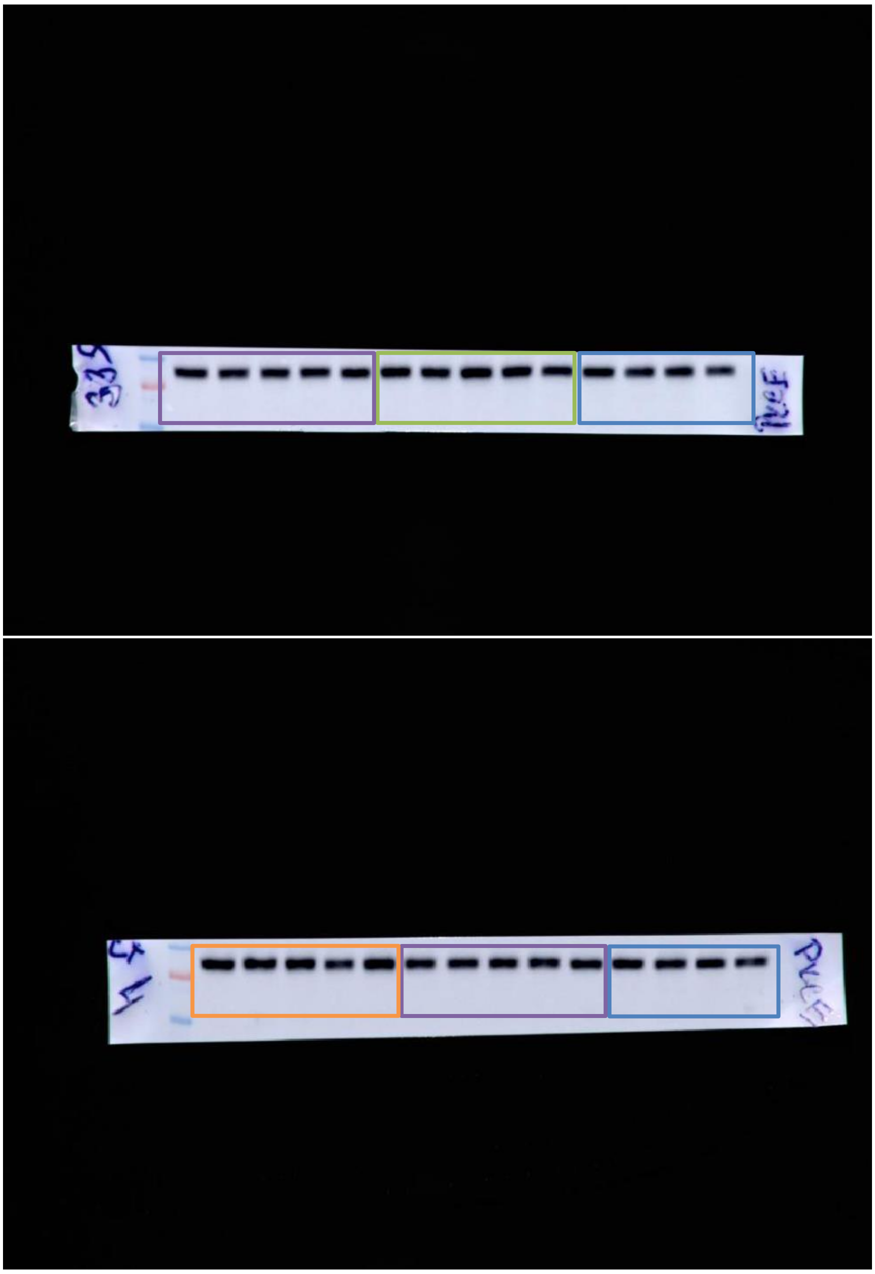

blue frame - HSD ACF
green frame - TGR ACF
violet frame - TGR(A1-7)3292
orange frame - TGR(A1-7)3292 ACF

LV

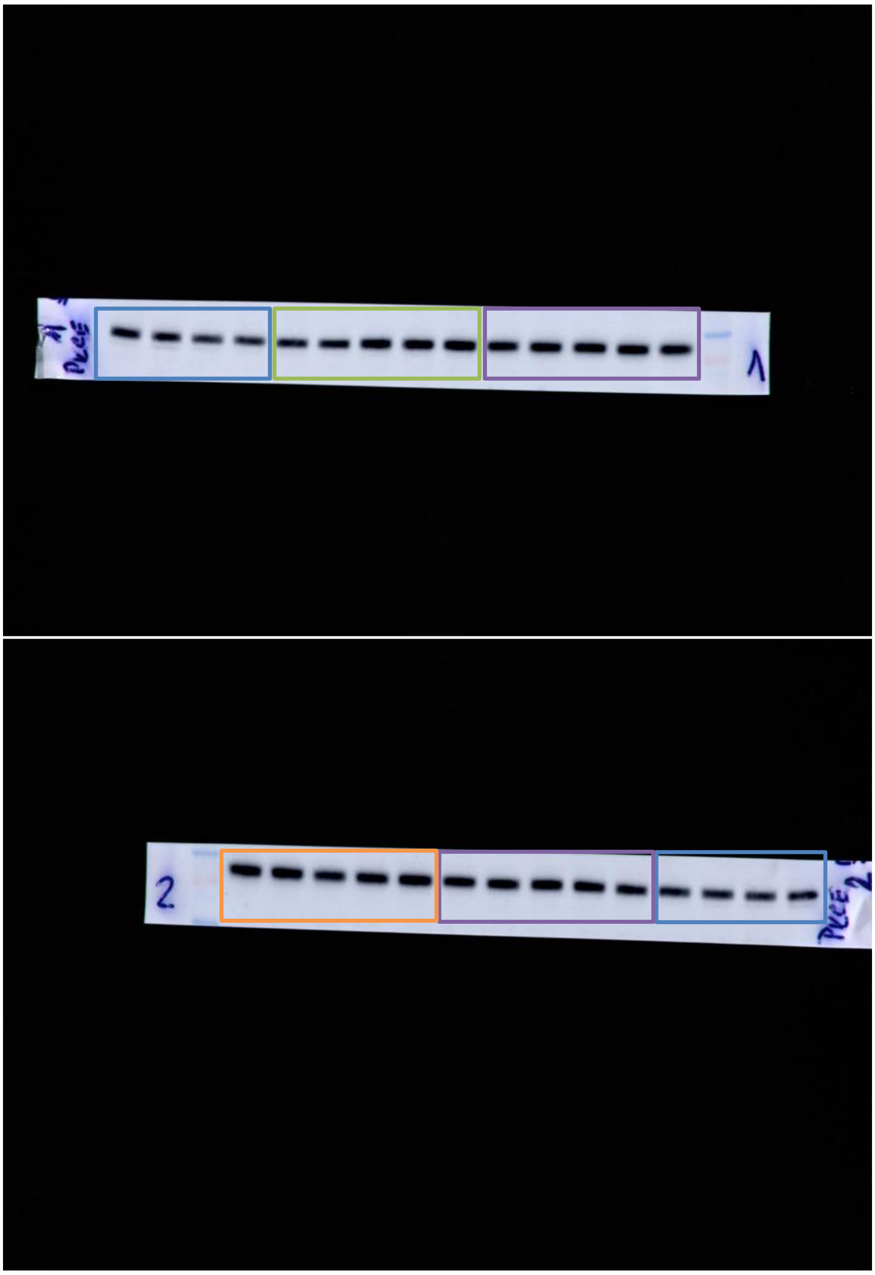

blue frame - HSD ACF
green frame - TGR ACF
violet frame - TGR(A1-7)3292
orange frame - TGR(A1-7)3292 ACF

pCx43

LV

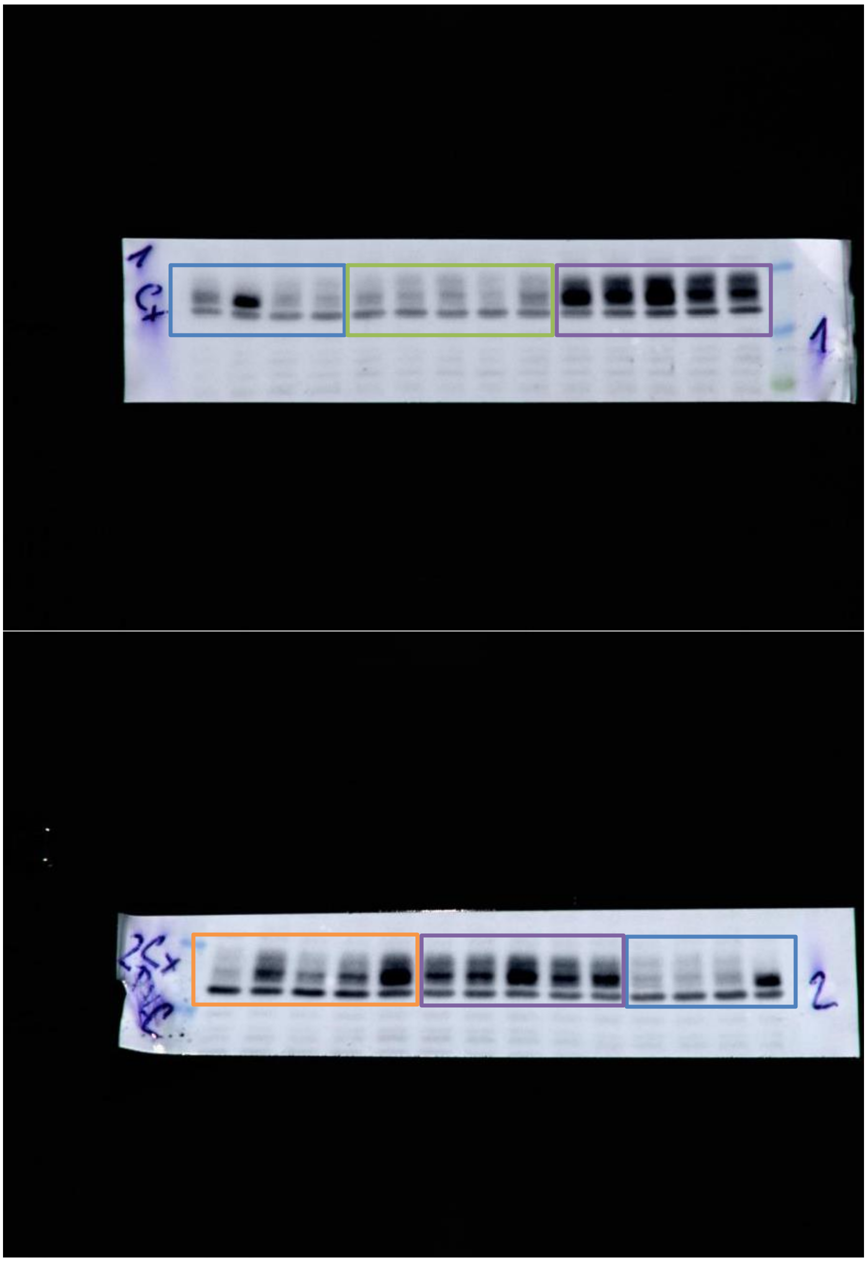

blue frame - HSD ACF
green frame - TGR ACF
violet frame - TGR(A1-7)3292
orange frame - TGR(A1-7)3292 ACF

RV

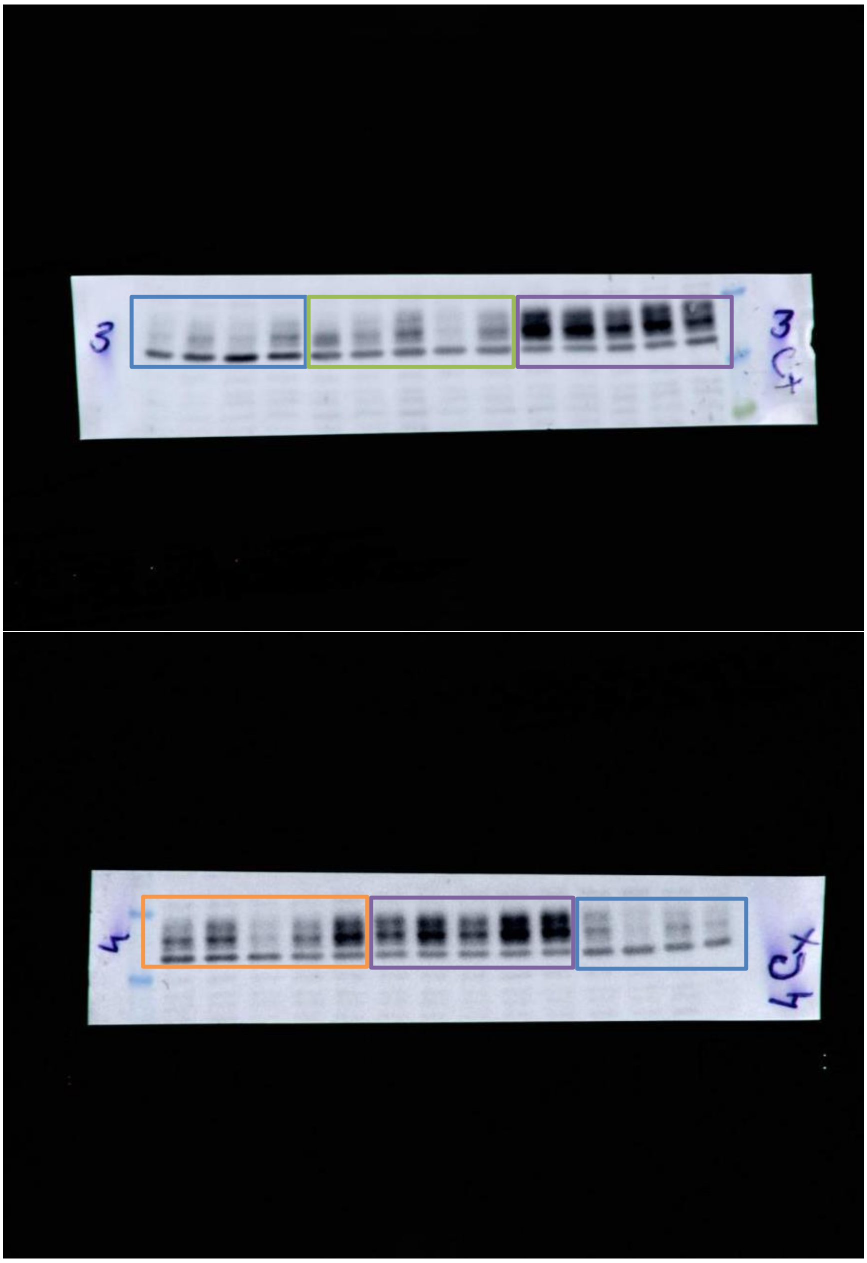

blue frame - HSD ACF
green frame - TGR ACF
violet frame - TGR(A1-7)3292
orange frame - TGR(A1-7)3292 ACF

Cx43

RV

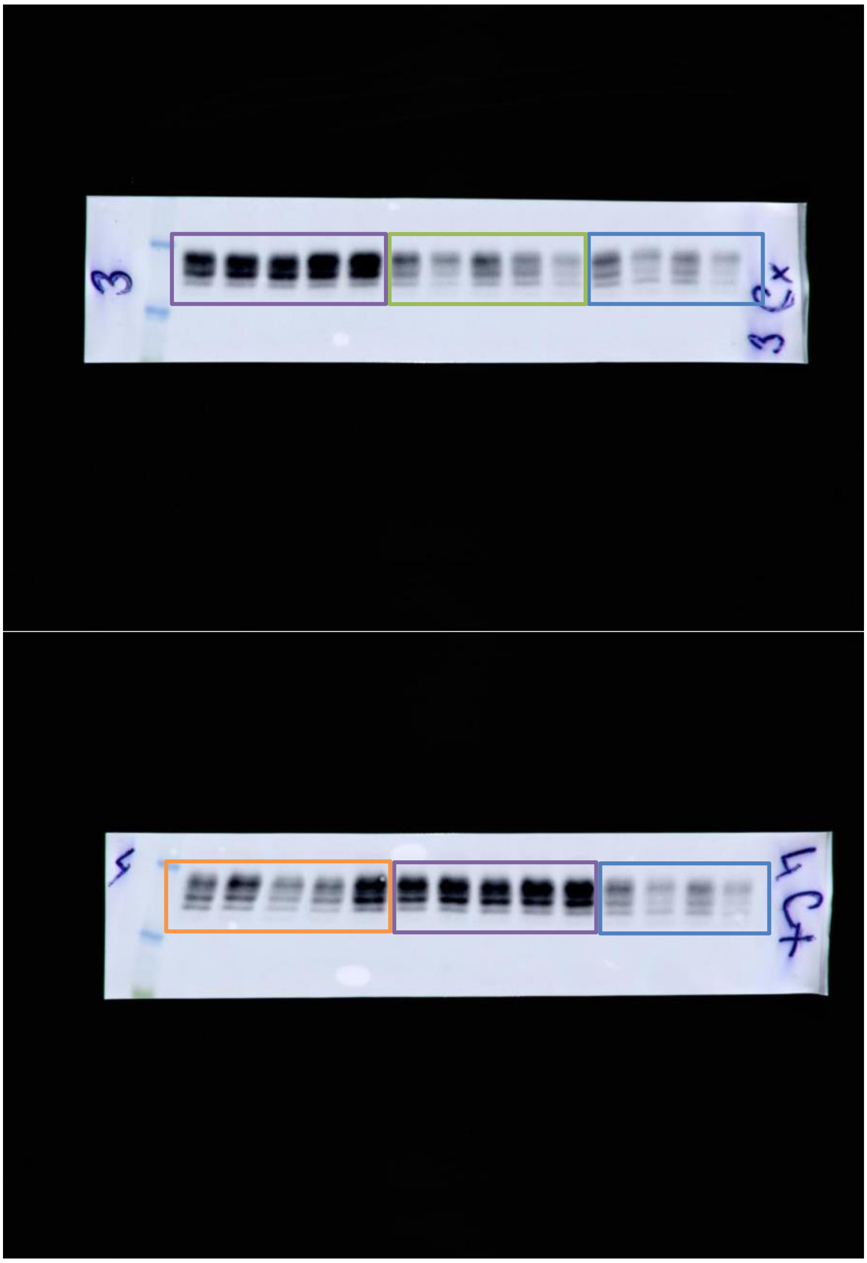

blue frame - HSD ACF
green frame - TGR ACF
violet frame - TGR(A1-7)3292
orange frame - TGR(A1-7)3292 ACF

LV

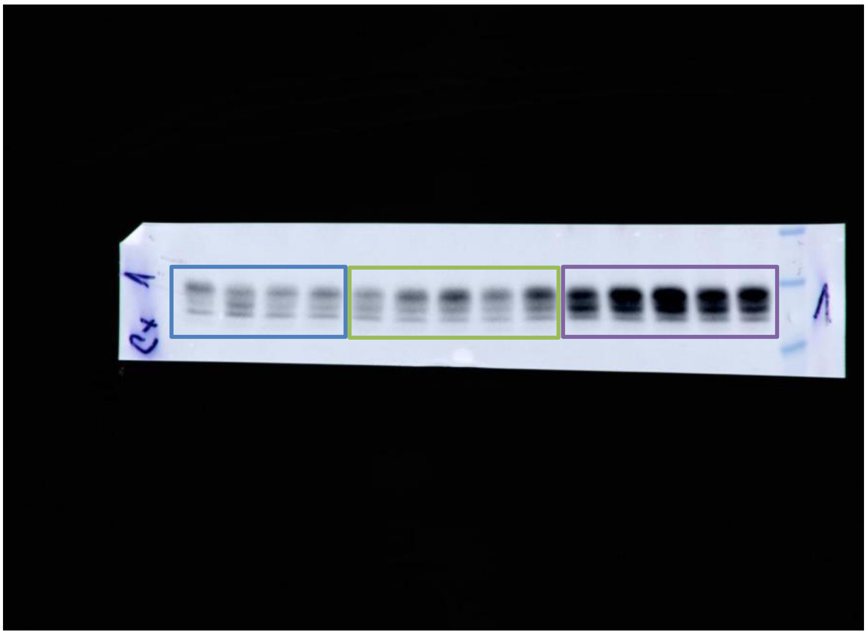

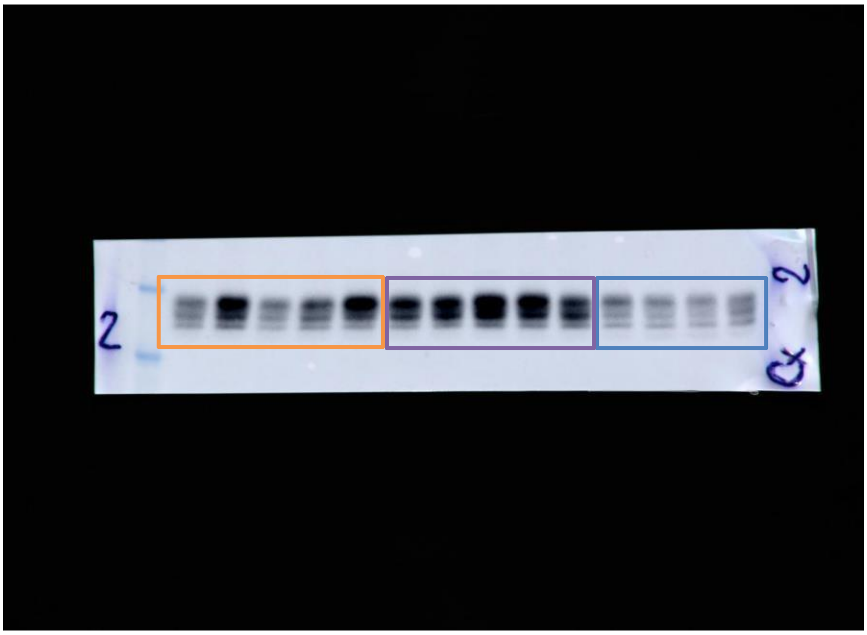

blue frame - HSD ACF
green frame - TGR ACF
violet frame - TGR(A1-7)3292
orange frame - TGR(A1-7)3292 ACF

MMP-2

LV

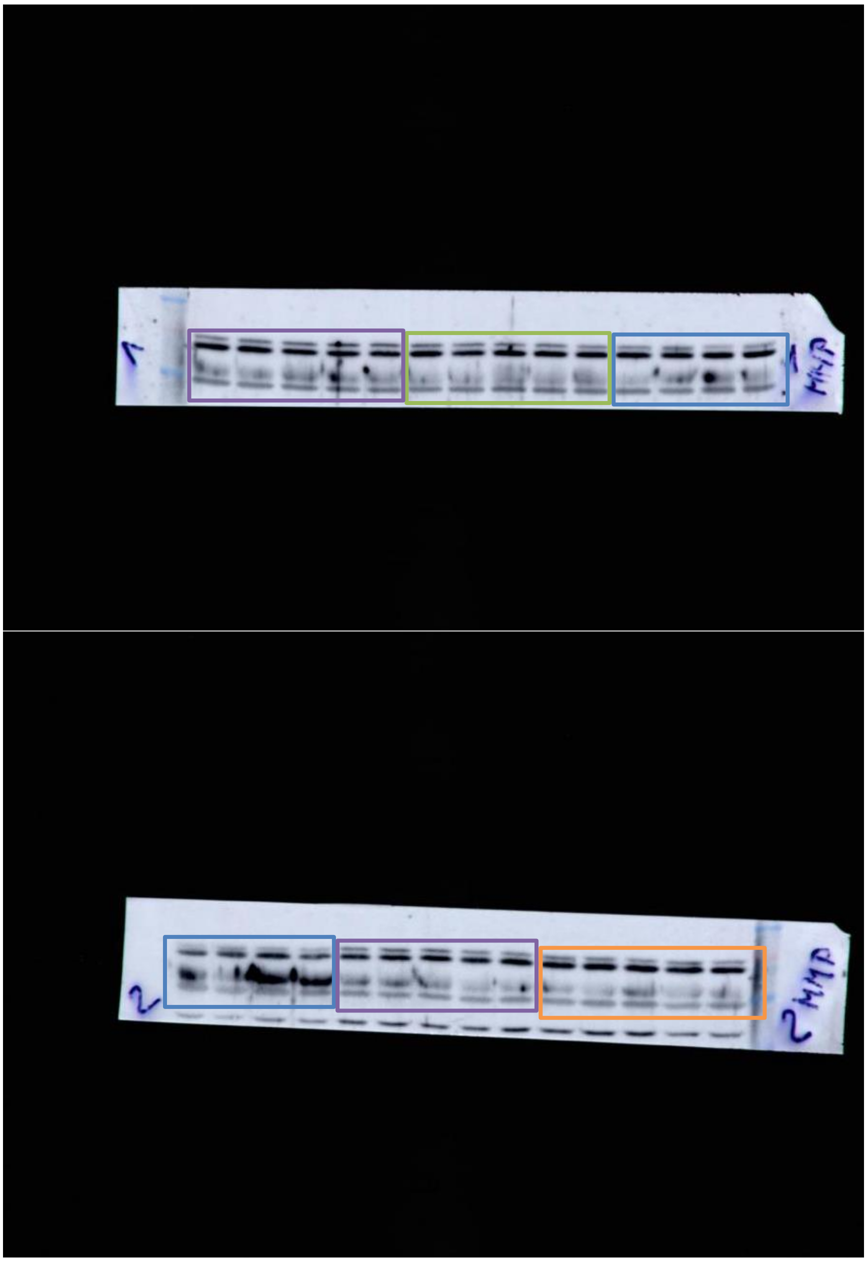

blue frame - HSD ACF
green frame - TGR ACF
violet frame - TGR(A1-7)3292
orange frame - TGR(A1-7)3292 ACF

RV

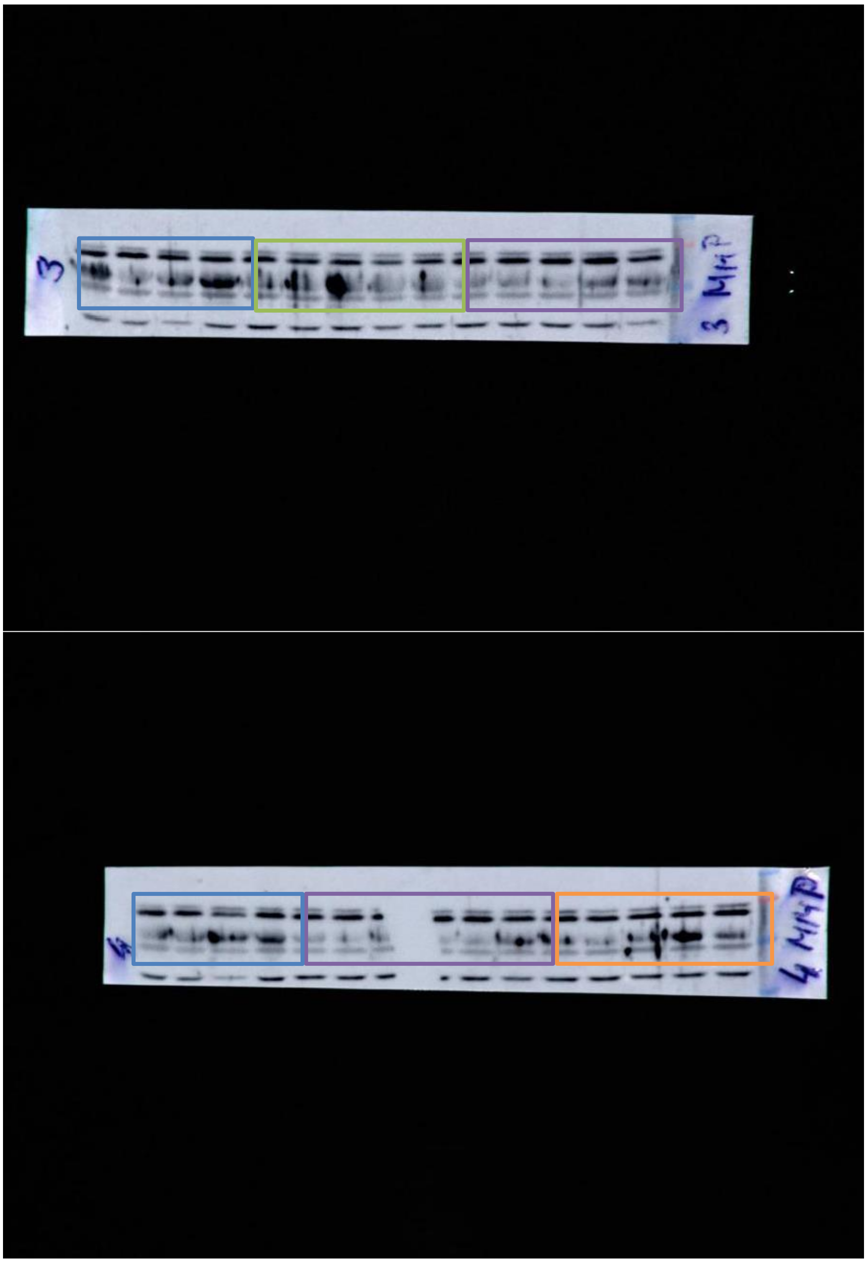

blue frame - HSD ACF
green frame - TGR ACF
violet frame - TGR(A1-7)3292
orange frame - TGR(A1-7)3292 ACF

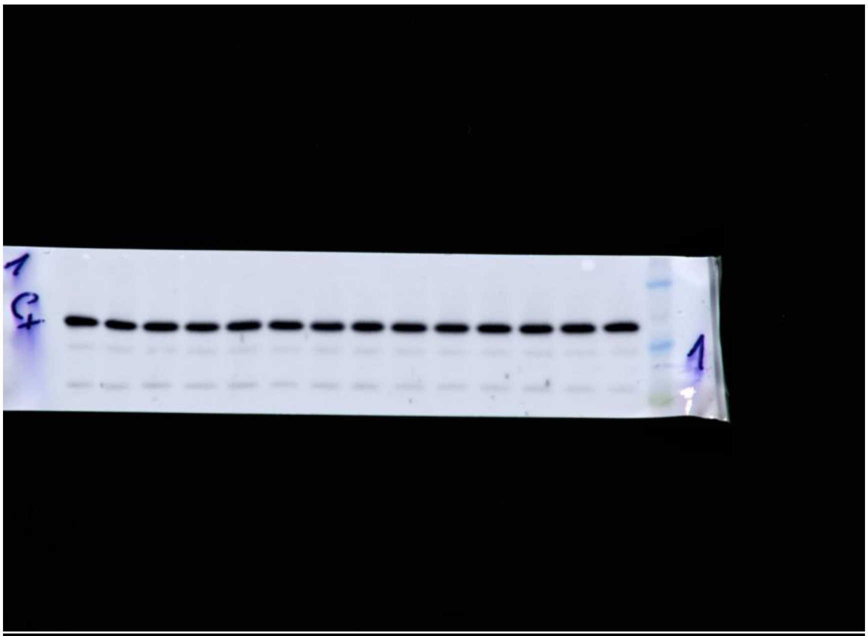
1
Ct
1

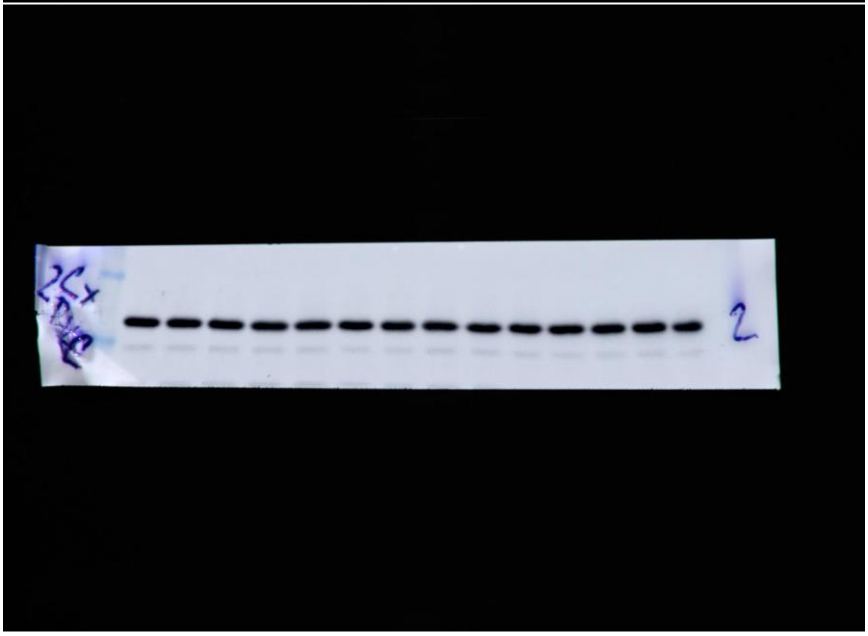
2Ct
2

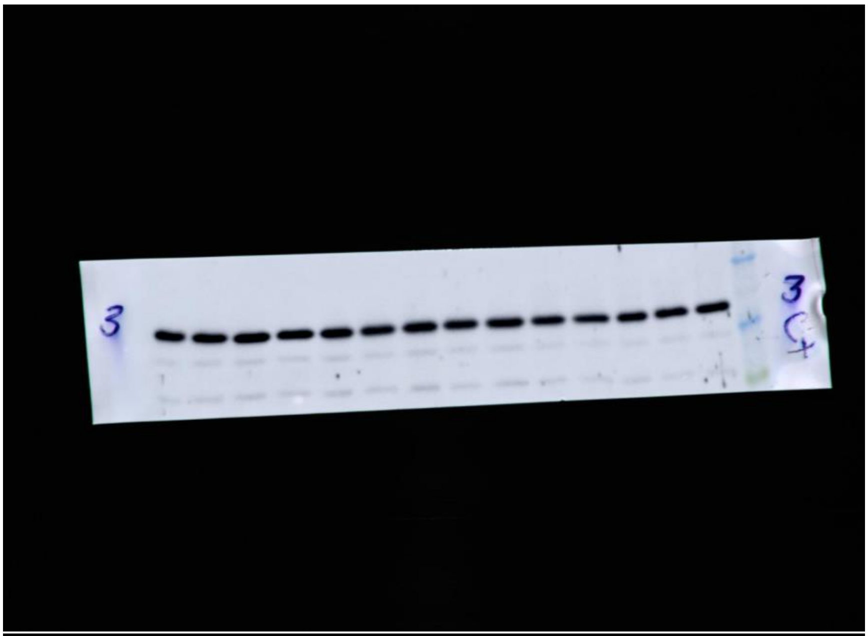
3

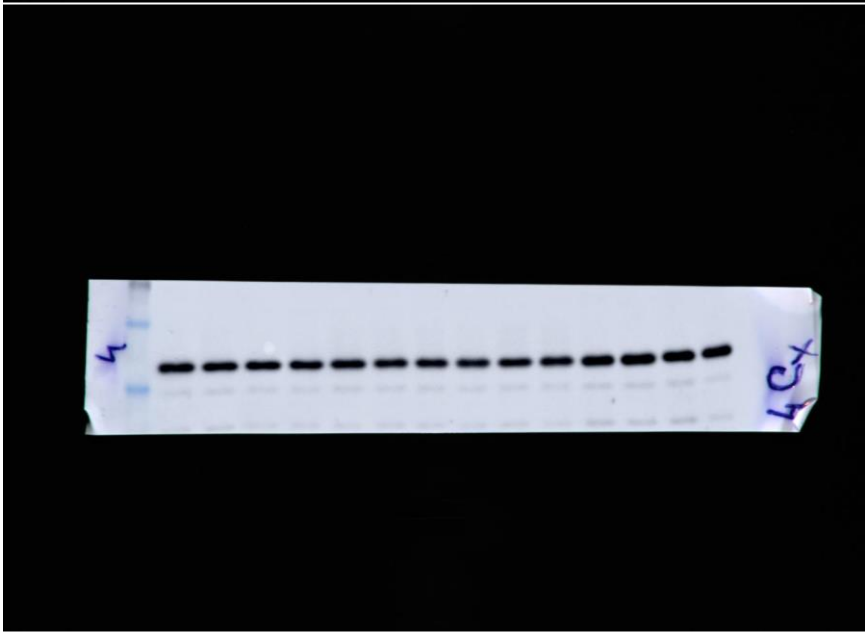
4

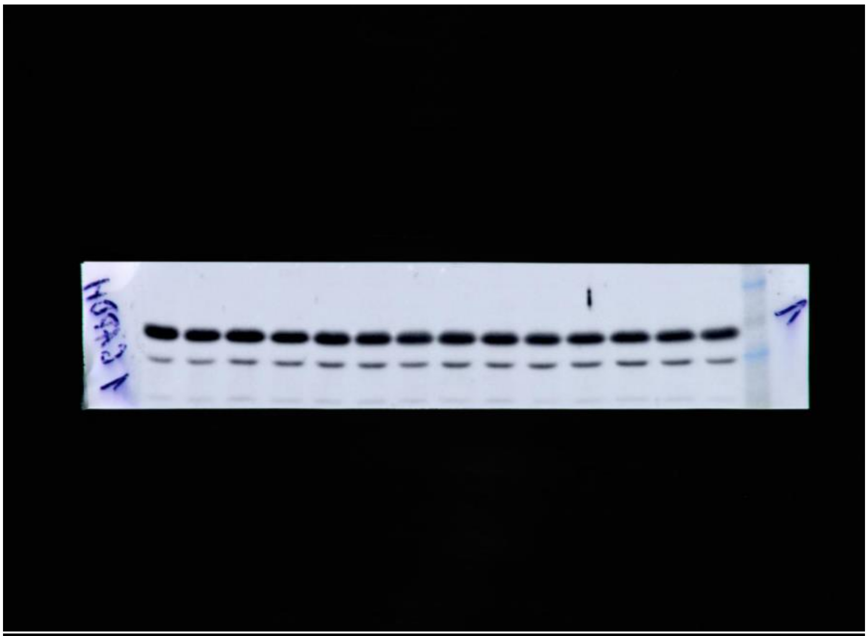
1 GAPDH
1

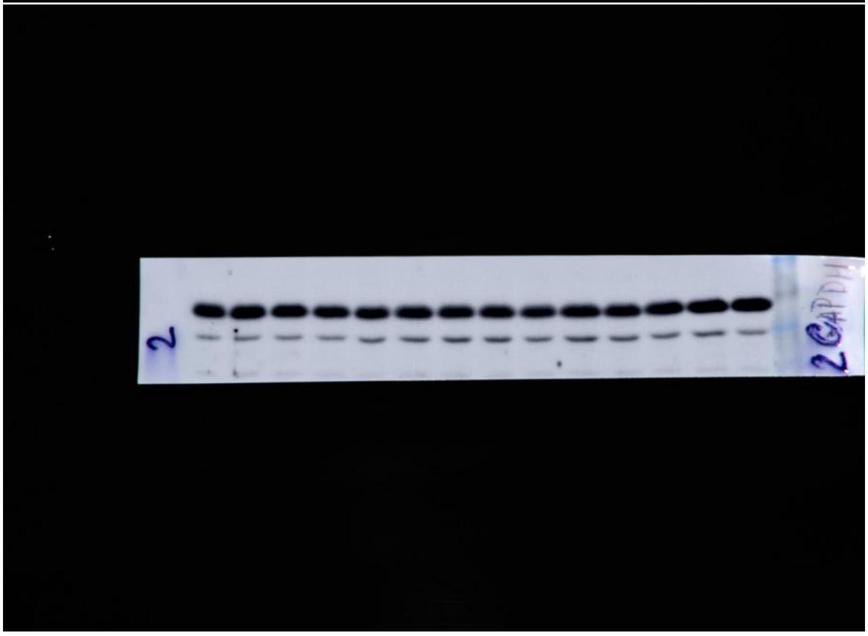
2
2 GAPDH

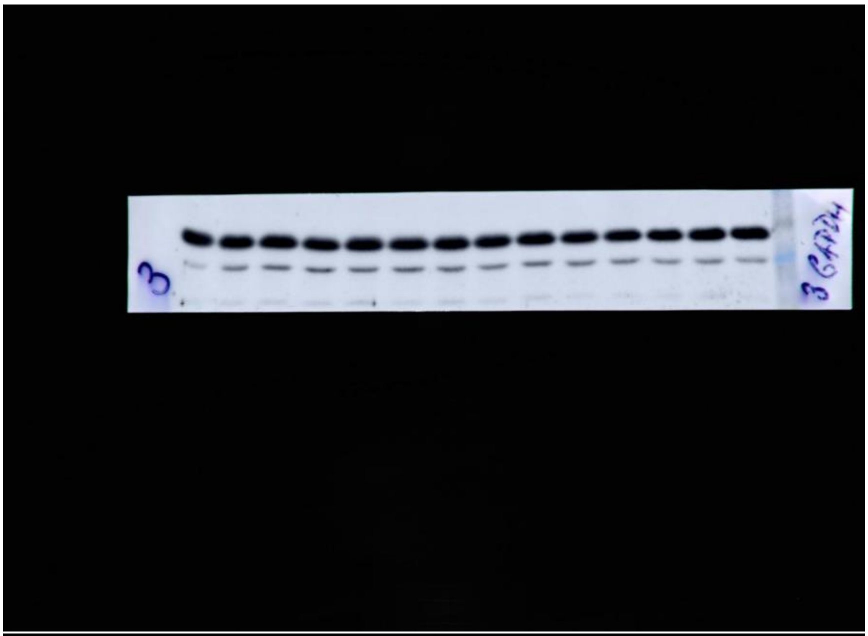
3
3 GAPDH

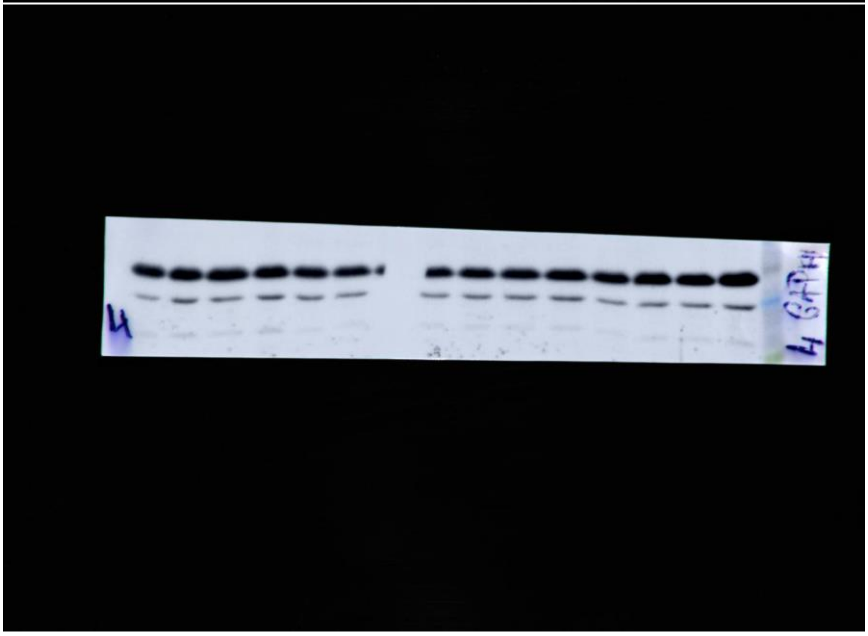
4
4 GAPDH

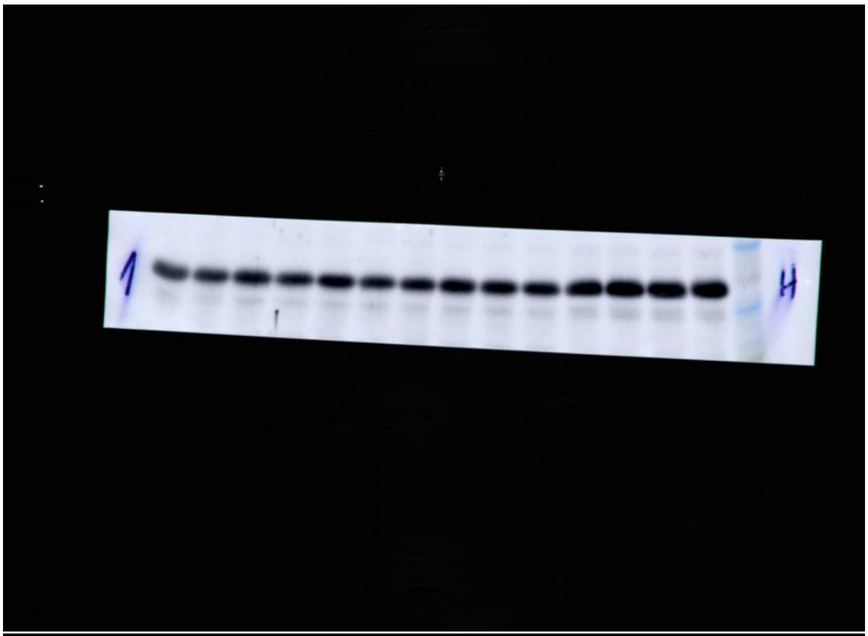
1
H

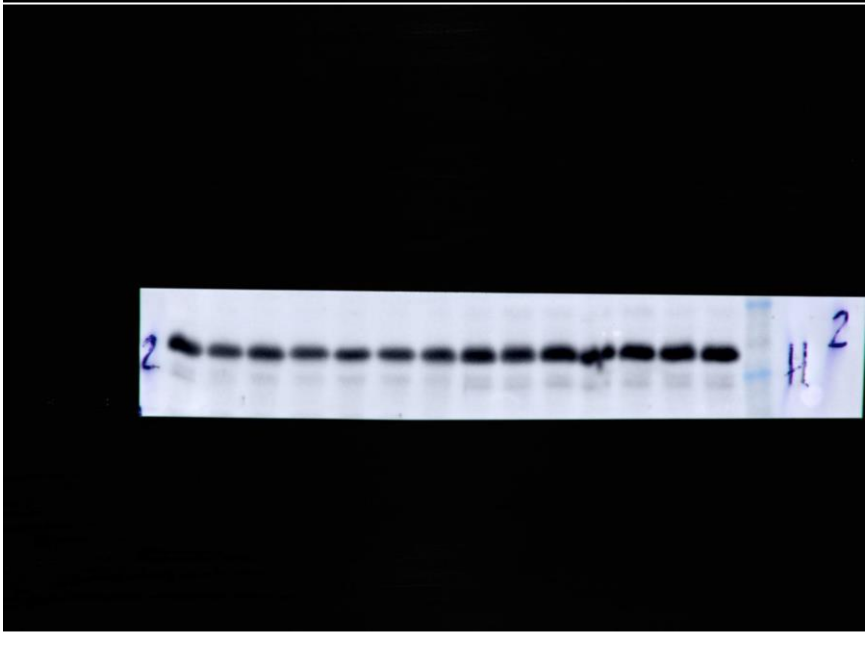
2
H
2

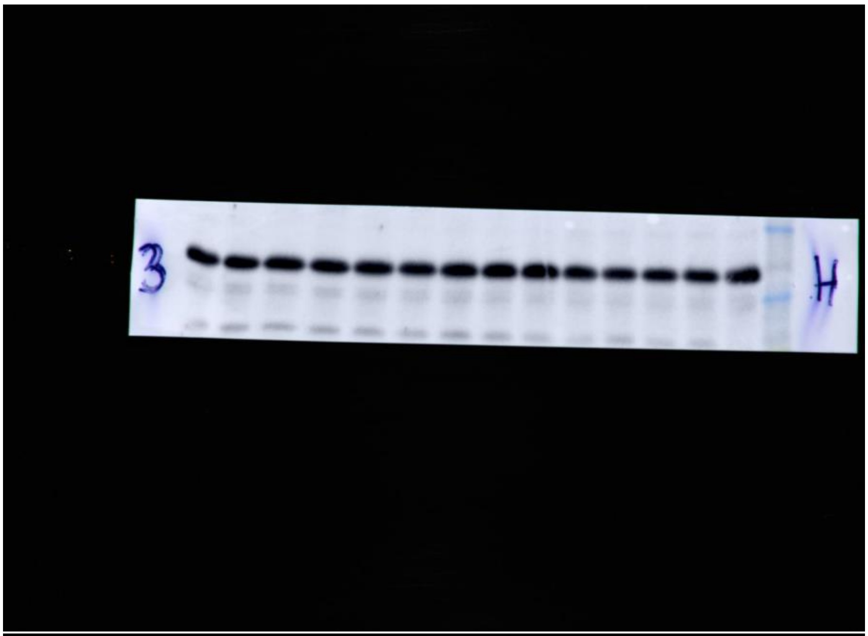
3
H

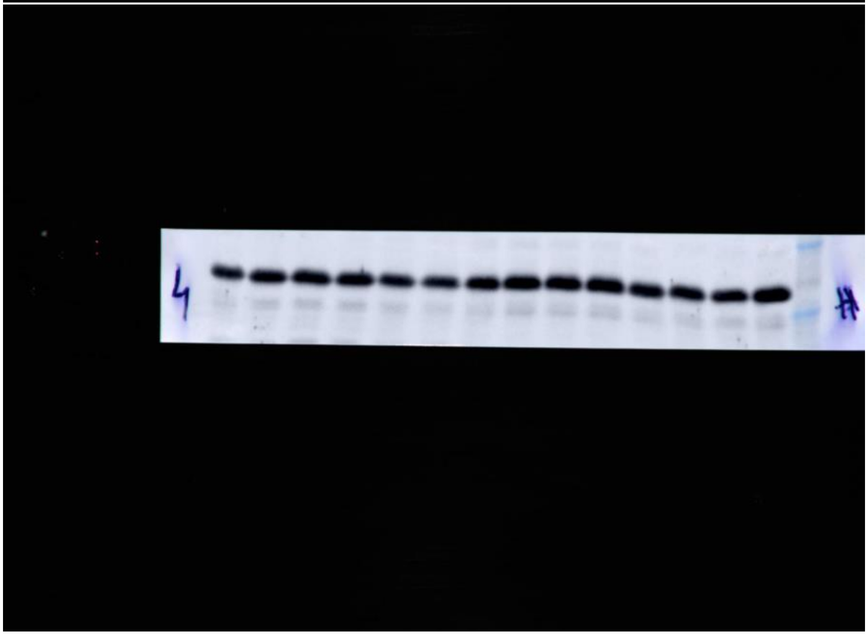
4
H

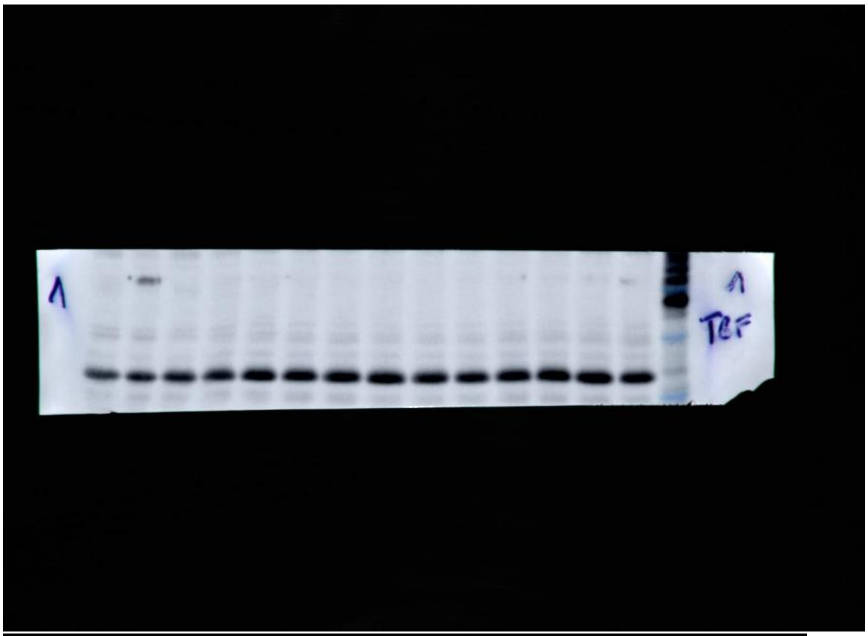

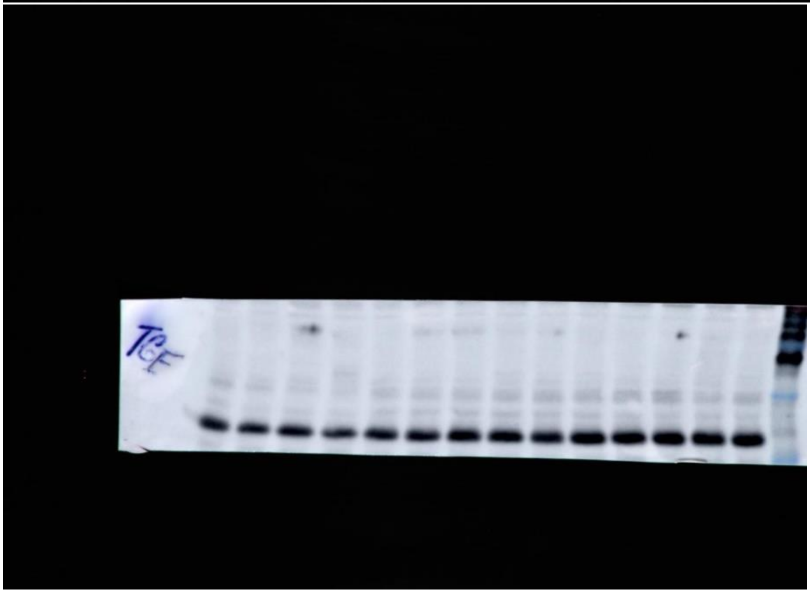

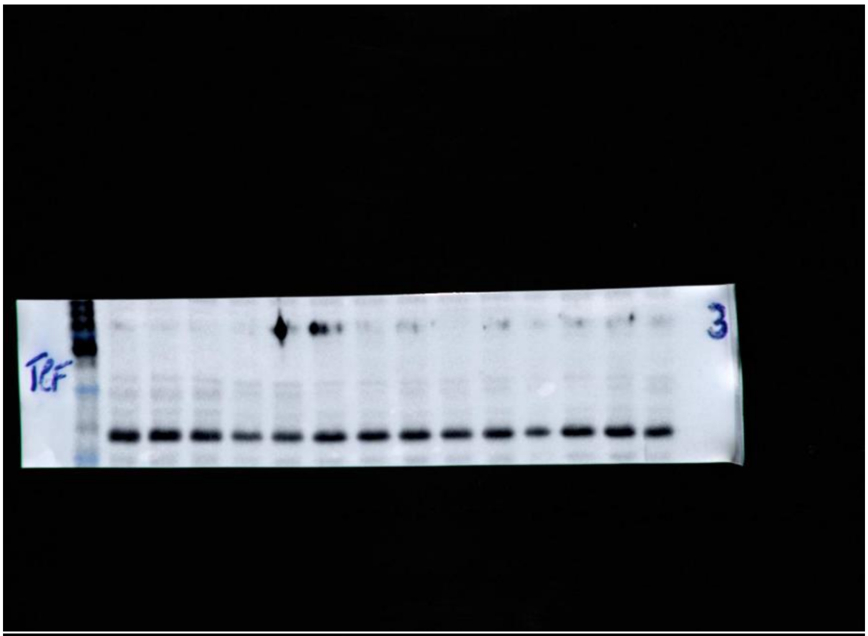
3

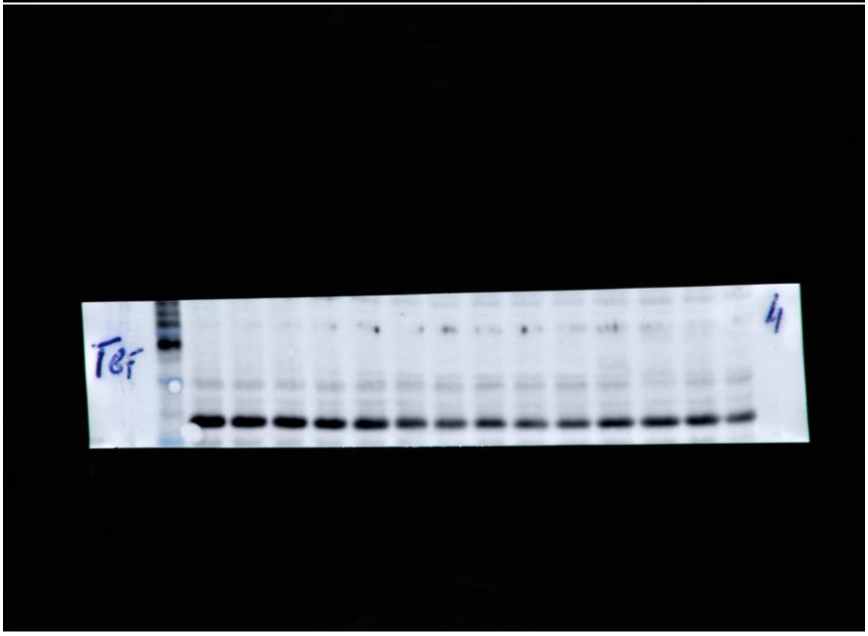
4
